# Comparison of the rhythm control treatment strategy versus the rate control strategy in patients with permanent or long-standing persistent atrial fibrillation and heart failure treated with cardiac resynchronization therapy - a pilot study of Cardiac Resynchronization in Atrial Fibrillation Trial (Pilot-CRAfT): study protocol for a randomized controlled trial

**DOI:** 10.1186/1745-6215-15-386

**Published:** 2014-10-04

**Authors:** Jan Ciszewski, Aleksander Maciag, Ilona Kowalik, Pawel Syska, Michal Lewandowski, Michal M Farkowski, Anna Borowiec, Tomasz Chwyczko, Mariusz Pytkowski, Hanna Szwed, Maciej Sterlinski

**Affiliations:** From the Second Department of Coronary Artery Disease, Institute of Cardiology, Warszawa, Poland

**Keywords:** Cardiac resynchronization therapy, Atrial fibrillation, Heart failure, Rhythm control, Cardioversion, Randomized controlled trial

## Abstract

**Background:**

The only subgroups of patients with heart failure and atrial fibrillation in which the efficacy of cardiac resynchronization therapy has been scientifically proven are patients with indications for right ventricular pacing and patients after atrioventricular junction ablation. However it is unlikely that atrioventricular junction ablation would be a standard procedure in the majority of the heart failure patients with cardiac resynchronization therapy and concomitant atrial fibrillation due to the irreversible character of the procedure and a spontaneous sinus rhythm resumption that occurs in about 10% of these patients.

**Methods/Design:**

Pilot-CRAfT is the first randomized controlled trial evaluating the efficacy of a rhythm control strategy in atrial fibrillation patients with cardiac resynchronization therapy devices. The aim of this prospective, single center randomized controlled pilot study is to answer the question whether the patients with cardiac resynchronization therapy and permanent atrial fibrillation would benefit from a strategy to restore and maintain sinus rhythm (that is ‘rhythm control’ strategy) in comparison to rate control strategy. The study population consists of 60 patients with heart failure and concomitant long-standing persistent or permanent atrial fibrillation who underwent a cardiac resynchronization therapy device implantation at least 3 months before qualification. Study participants are randomly assigned to the rhythm control strategy (including electrical cardioversion and pharmacotherapy) or to the rate control group whose goal is to control ventricular rate. The follow-up time is 12 months. The primary endpoint is the ratio of effectively captured biventricular beats. The secondary endpoints include peak oxygen consumption, six-minute walk test distance, heart failure symptom escalation, reverse remodelling of the heart on echo and quality of life.

**Trial registration:**

NCT01850277 registered on 22 April 2013 (ClinicalTrails.gov)

## Background

The heart failure (HF) patients with concomitant atrial fibrillation (AF) make up a significant subgroup of patients eligible for cardiac resynchronization therapy (CRT) but their treatment remains problematic.

The prevalence of AF as well as chronic HF is significant and it is still growing. It has been estimated that 1 to 2% of population suffers from AF - the most common form of sustained arrhythmia [1]. The prevalence of HF in general population is 1 to 2% and rises with age, reaching >10% in a subpopulation of subjects aged 70 years or older [2]. The coexistence of AF and HF is very frequent. Both illnesses may negatively influence and promote each other [1]. The coexistence of AF in HF patients is related to the severity of heart failure. The prevalence of AF in New York Heart Association (NYHA) class I is about 5%, reaching 25 to 50% in NYHA III/IV subgroups [3, 4]. About 23% of patients undergoing a CRT device implantation in Europe have AF [5]. With population ageing, estimated growth in the prevalence of AF and HF and broad label of CRT implantation, the issue of proper treatment of CRT patients with AF becomes crucial.

Nevertheless, the CRT in patients with AF encounters many problems and its beneficial effect in this group of patients is not fully established. The indications for CRT arise from the evidence coming from the studies where AF patients were absent or barely present. The only subgroup of patients with AF in which beneficial effect of CRT is scientifically proven (that is proven in randomized controlled trails (RCTs)) are the patients with concomitant indications for right ventricular pacing [6, 7]. As a result, the present indications for the CRT in patients with concomitant AF stem from the recommendations for those in sinus rhythm (SR), having a level of evidence C in IIb class [2]. Moreover, there is no sufficient data evaluating the CRT impact on mortality in the AF patients and an emphasis is put on the lack of trials comparing the rhythm control versus rate control strategy in this group of patients [2, 5].

Contrary to the widening range of patients with HF suitable for CRT, the evidence is growing that the benefit of resynchronization in the subgroup of patients with AF is reduced. The initial meta-analyses of the studies comparing the efficacy of CRT in patients with AF versus SR suggested similar effect of response to CRT in the AF group in terms of NYHA functional class or ejection fraction and lower effect on the exercise tolerance improvement measured by means of six-minute walk test (6MWT) [8, 9]. The recent and larger meta-analysis has shown however, that the patients with AF have higher mortality and higher rate of non-response to CRT than their SR counterparts [10]. Whatever the results of those systematic reviews, it should be mentioned that the vast majority of the data included in those analysis has been derived from observational studies - not randomized trials, which are not available.

The lower effectiveness of CRT in AF patients is explained by the irregular and unpredictable character of ventricular beats in this arrhythmia which lowers the ratio of effectively biventricular paced captured beats (BiVp%). This hypothesis is corroborated by studies of AF patients on CRT undergoing atrioventricular junction ablation (AVJA), which enables the BiVp% to be close to 100%. According to the registries, the CRT efficacy in patients with AF undergoing AVJA is similar to that in patients in SR, whereas the patients with AF but no AVJA seem not to benefit from resynchronization at all, even if BiVp% exceeds 85% [3, 11]. As a result, the estimated BiVp% that guarantees the CRT efficacy is ≥95% [11] or even ≥98 to 99% [5, 12, 13]. The most recent ESC guidelines put an emphasis on the achievement of biventricular capture as close as possible to 100% and - unless achieved - on performing AVJA [5]. Nevertheless, the significant group of AF patients with already-implanted CRT devices present with this goal not sufficiently met.

Although AVJA seems to provide the efficacy of CRT [2, 5, 7, 14], it is unlikely that performing an iatrogenic III° atrioventricular block will be recommended in all patients with AF undergoing CRT implantation. Such an approach would generate an enormous group of pacemaker-dependent patients - a potentially dangerous situation. Moreover, performing an AVJA seems questionable, bearing in mind the spontaneous SR resumption that occurs in 10 to 25% of the CRT patients with permanent/long-standing persistent AF [15, 16]. This phenomenon may occur even a few years after the implantation of CRT device [16]. Due to the limitations of the AVJA, the strategy of SR resumption and its maintenance seems to be a reasonable option in the group of CRT patients with AF.

To the authors’ best of knowledge, a randomized, prospective trial assessing the influence of rhythm control strategy on CRT efficacy in AF patients has not yet been performed. The only trial upon this issue was the non-randomized, cohort study of Turco *et al*. [17], which has shown a positive effect on end-diastolic volume in the rhythm control group, whereas the influence on ejection fraction was comparable between the rhythm and the rate control groups.

The promising results of the rhythm control strategy were also achieved in the PABA-CHF trial which compared pulmonary vein isolation (PVI) versus the *ablate and pace* strategy (that is performing an AVJA + CRT-D (CRT with defibrillator function) implantation) in HF patients with paroxysmal or persistent/permanent AF [18]. During the 6-month observation the patients who underwent PVI had longer 6MWT distance, higher ejection fraction and they claimed better quality of life. The effect was even more profound in the permanent/persistent AF subgroup. The trial did not prove any favorable impact of rhythm control strategy on mortality and showed a higher hospitalization rate in this group; however, it analyzed the overall group HF and AF patients, not the subgroup meeting the current CRT-D implantation criteria and compared the PVI alone (that is not combined with CRT) has in comparison to the patients treated with CRT + AVJA.

## Methods/Design

### The aim of the study

The aim of the study is to answer the question whether the patients with permanent or long-standing persistent AF on cardiac resynchronization therapy due to heart failure will benefit from the treatment strategy to resume and maintain sinus rhythm in terms of achievement of adequate BiVp%. An additional goal of the study is the identification of potential factors predicting the efficacy/the lack of benefit of the rhythm control strategy. As it is a pilot study, its other goal is to collect data which would enable the authors to design another, larger study, which would have a power to examine the influence of a rhythm control strategy versus rate control strategy on the mortality and morbidity of HF in this group of patients.

### Study scheme

The Pilot-CRAfT study is a single-center, open, prospective, randomized controlled trial. The trial enrolls 60 patients with HF and concomitant permanent or long-standing persistent AF who underwent CRT implantation at least three months before the enrollment in the Second Department of Coronary Artery Disease of the Institute of Cardiology in Warsaw, Poland. The inclusion and exclusion criteria are listed in the Table 1. Before the enrollment each participant will give written, informed consent.

**Table 1:** **The inclusion and the exclusion criteria for the Pilot-CRAfT study**

Inclusion criteria:	
1)	Permanent or long-standing persistent AF (definitions according to the latest ESC guidelines on AF)
2)	At least three months after the procedure of a CRT device implantation
3)	A CRT device with the presence of a right atrial electrode
4)	Age: ≥18 years old
5)	Effectively biventricular paced captured beats percentage <95%
6)	Effective therapy with oral anticoagulants for at least three weeks
7)	Written informed consent
Exclusion criteria:	
1)	Reversible causes of AF
2)	Significant valve disease
3)	Advanced AV block (including: AVJA)
4)	Contraindications to amiodarone (hyperthyroidism, non-compensated hypothyroidism, drug intolerance, QT >460 ms for men, QT >450 ms for women)
5)	Long-QT syndrome
6)	Cardiac transplantation in six months
7)	Life expectancy less than one year
8)	LA diameter larger than 6 cm
9)	Heart failure decompensation within 48 hours before the qualification
10)	Chronic dialysis
11)	History of alcohol abuse
12)	Pregnancy or lack of effective contraceptive therapy (in case of females in the reproductive age)
13)	Participation in other clinical trial

AF, atrial fibrillation; AV, atrioventricular; AVJA, atrioventricular junction ablation; CRT, cardiac resynchronization therapy; ESC, European Society of Cardiology; LA, left atrium.

### Principles of the management of the patients (treatment strategies)

The enrolled patients are randomly assigned to one of the two groups (in a 1:1 ratio). In the first group, a strategy to restore and maintain SR is implemented (that is rhythm control strategy - the active treatment group). In the second group, a strategy to slow ventricular rate will be implemented (that is rate control strategy - the control group).

#### Rhythm control group

The basic method to restore and maintain sinus rhythm comprises a combination of pharmacological antiarrhythmic treatment and external electrical cardioversion (EEC). The attempts to restore SR must not be performed unless the patient has been treated effectively with oral anticoagulants for at least three weeks.

The pharmacological treatment consists of amiodarone administered orally. The loading dose of amiodarone is up to 600 mg daily for the first 4 weeks. Then, a maintenance dose of 200 mg/daily is administered (a dose may be adjusted at discretion of treating physician). The loading dose may be modified or omitted, especially, if a patient had been given amiodarone before the enrollment. If any contraindications to amiodarone occur during the treatment, it must be discontinued promptly. The use of other antiarrhythmic agents is possible unless they are contraindicated.

A basic tool to restore SR in the trial is an EEC. The first EEC should be performed after the complete loading dose of amiodarone has been administered (unless SR is restored spontaneously). Up to three shocks during one cardioversion are allowed. The amount of the energy delivered is left to discretion of a physician performing the EEC. If AF recurs, the patient should undergo the next EEC as soon as possible but preserving the safety time margins of effective anticoagulation period (at least three weeks). The maximal number of EEC procedures is three and the third one is possible only if AF recurs no earlier than three weeks after the second EEC and SR resumption is related to alleviation of HF symptoms. If SR resumption or its maintenance is impossible or AF recurs after the third EEC, a strategy of *rhythm control* is discontinued and a *rate control* strategy is implemented.

In patients undergoing the *rhythm control strategy* treatment, a procedure of ablation of AF substrate (that is PVI) is possible. The decision to offer the patient an ablation must be made collectively at the discretion of the therapeutic team. Routinely in the trial, a PVI procedure is allowed and offered to patients responding to EEC after the second recurrence of AF.

#### Rate control group - control group

The treatment goal in the *rate control* group (that is the control group) is to slow and control ventricular rate by means of pharmacological antiarrhythmic agents and AVJA as to achieve satisfactory BiVp%. The pharmacotherapy should include negative chronotropes and negative dromotropic drugs such as beta-blockers, digitalis and amiodarone (the use of other, less popular agents, is also possible). It should be consistent with the current guidelines of treatment of HF and AF. The choice of the agents and its’ dosage is left to discretion of the treating physician. An AVJA procedure in this group of patients is possible but not obligatory. The decision to perform AVJA is left to the discretion of the treating physician. An effective anticoagulant treatment is essential in both groups.

### Follow-up

The follow-up period is 12 months. The patients are scheduled for control visits every three months. Every control visit consists of: a standard review of a device and treatment; an assessment of the actual BiVp%; the questions concerning the occurrence of the analyzed endpoints and a standard 12-lead ECG. Additionally, on the qualification visit, and control visits after 3 and 12 months, a 6MWT, an echocardiograph and a cardiopulmonary exercise test (CPX) are performed and the patients are asked to fill the questionnaire about their quality of live, that is the Minnesota Living with Heart Failure Questionnaire (MLHFQ), © University of Minnesota [19, 20], (MLHF contact information and permission to use: MAPI Research Trust, Lyon, France. Email: PROinformation@mapi-trust.org - Internet: http://www.mapi-trust.org and University of Minnesota. Email: mlhfq@umn.edu - Internet http://www.mlhfq.org). Moreover, a thyroid-stimulating hormone (TSH) level control is performed on the qualification visit and during visits after six and twelve months.

### Endpoints

All of the endpoints will be assessed in two timeframes: at three months and one year from the baseline examination timeframe.

The primary endpoint is an overall BiVp% during the one-year study period (that is a mean BiVp% through the whole follow-up period). The ‘triggered’ paced beats are not treated as effective and are excluded from the analysis.

The secondary endpoints are listed in the Table 2.

**Table 2:** **Secondary endpoints of the Pilot-CRAfT trial**

	Secondary endpoints:
BiVp%:	
•	BiVp% change from baseline.
Physical competence:	
•	6MWT distance
•	Peak oxygen uptake (peak VO_2_) in CPX
•	NYHA functional class
The echocardiographic assessment of the reverse remodelling:	
•	Increase in left ventricle ejection fraction (LVEF)
•	Decrease in left ventricle diastolic diameter and volume (LVEDD, LVEDV)
•	Decrease in left ventricle systolic diameter and volume (LVESD, LVESV),
•	Decrease in size of the left atrium (LA)
•	Decrease of the severity of mitral insufficiency
Quality of life:	
•	Measured with the Minnesota Living with Heart Failure Questionnaire
Clinical endpoints:	
•	Unplanned hospitalizations due to heart failure exacerbations
•	Death
•	Cardiovascular death
•	Hospitalizations due to cardiovascular causes
•	Stroke/TIA
•	A combined endpoint including unplanned hospitalizations due to cardiovascular causes or death
Heart arrhythmias:	
•	AF - using *mode switch* to measure time of AF with fast ventricular rate
•	Ventricular tachycardia including *torsade de pointes* tachycardia and ventricular fibrillation
In the CRT-D subgroup:	
•	Number of adequate and inadequate shocks (separately)
•	The prevalence of electrical storm
Adverse events:	
•	Pharmacotherapy related
•	Device related
•	Cardioversion related
•	AVJA related

The clinical endpoints, the occurrence of heart arrhythmias and adverse events are collected for safety measures. All of the endpoints will be assessed at three-months and one-year from the baseline timeframes.

6MWT, six-minute walk test; AF, atrial fibrillation; AVJA, atrioventricular junction ablation; BiVp%, effectively biventricular paced captured beats percentage; CPX, cardiopulmonary exercise test; CRT-D, cardiac resynchronization therapy with defibrillator function; TIA, transient ischemic attack.

Based on the results of the primary endpoint, the authors plan to perform a preliminary identification of the factors predicting the response to the *rhythm control* strategy.

### Methodology of additional examinations and tests

#### Device control

Device interrogation is performed on every control visit. It should include: a standard battery and lead control, analysis of the histograms, alerts (especially arrhythmic), history of the ‘mode switch’ episodes (in order to evaluate AF burden) and BiVp% (measured from the last control visit). In case of CRT-D devices, the episodes of arrhythmia as well as the number of adequate and inadequate high-voltage intervention should be registered. If required, necessary modifications in the identification and treatment of ventricular arrhythmias should be made.

Moreover, during the visit a review of pharmacological treatment is performed, including analysis of its efficacy, validity, and occurrence of adverse effects. If required, necessary modifications in the pharmacological treatment, an eventual decision to change the *rhythm control* for the *rate control* or a decision to perform AVJA should be made. If an intervention of a device occurs, the additional device control should be performed.

#### Echocardiography

Participants will undergo an echo test (Philips Matrix iE33, Philips Ultrasound, Bothell, WA, USA) performed with a general assessment of morphology and function of the heart.

Echocardiography will include the evaluation of:

- Cardiac dimensions (including diameter, area and volume of left atrium)
- Left ventricular global systolic function (EF% and volumes, dp/dt), left ventricular regional function (extent and localization of infarct scar) and left ventricle diastolic function
- Type of diastolic dysfunction, diastolic pressure in the left ventricle
- Presence and severity of valvular diseases (particularly mitral regurgitation)
- Evaluation of cardiac dyssynchrony
- Intraventricular dyssynchrony parameters ('septal flash' and septal to posterior wall motion delay, septal to lateral time to peak systolic point from R-wave on electrocardiogram (Ts) delay)
- Atrioventricular dyssynchrony: diastolic filling ratio (LVFT/RR)
- Interventricular dyssynchrony (difference between left ventricular and right ventricular pre-ejection time in pulsed-wave Doppler and/or a delay between the onset of systolic motion in the basal right ventricular free wall versus the most delayed basal LV segment in tissue Doppler)

In patients with sinus rhythm, an AV interval will be optimized (unless optimization had been performed previously).

#### Cardiopulmonary exercise test

CPXs on a treadmill (Marquette T2000, GE Healthcare, Little Chalfont, UK) with standardized modified Bruce or Naughton exercise protocols will be applied. Breath-to-breath respiratory gas analysis will be carried out with Vmax Encore 29c equipment (Viasys Healthcare, San Diego, CA, USA). An exercise test will be carried out until maximal patient’s exertion, maximal heart rate or dangerous ventricular arrhythmia occurs. The following parameters will be monitored during CPX:

- Heart rate, arterial blood pressure, ST segment changes
- Peak oxygen uptake (peak VO_2_, ml/kg/minute, l/minute)
- Peak VO_2_ as percentage of normalized VO_2_ in given population
- Peak carbon dioxide exhalation (peak VCO_2,_ l/minute)
- Oxygen pulse (O_2_ pulse, ml/heart rate)
- Maximal minute ventilation (VE, l/minute)
- Breathing reserve (BR): maximal voluntary ventilation/maximal exertional ventilation × 100%
- Heart rate reserve (HRR): maximal HR/peak exertional HR × 100%
- Anaerobic threshold level (ml/kg/minute)
- Maximal CO_2_ equivalent: VE/VCO_2_ max
- VE/VCO_2_ slope and VE/VO_2_ slope
- Subjective dyspnea level (according to Borg scale from 1 to 5, where 5 is maximal dyspnea)

Exertional level will be assessed subjectively by patients in Borg scale (6 to 20, where 6 means no exertion, 20 - maximal exertion).

## Statistical methods

### Sample size

The calculation of a sample size was made based on the estimated results in the groups for the primary endpoint (BiVp%) and the following assumptions: 1) the mean difference of BiVp% between groups would be 8% and standard deviation (SD): 15%; 2) probability of a type I error: 0.05; 3) probability of a type II error: 20%; 4) the type of test: Student’s two-sample *t*-test; 5) drop-out rate: 8%.

A power analysis revealed that a minimum of 30 patients per group will be required to assure at least 80% power for detecting the anticipated between-group differences in BiVp% and to compensate for the expected drop-out rate.

### Randomization

Randomization will be provided by independent statistician using SAS 9.2 software (SAS Institute Inc., Cary, NC, USA). Permuted block randomization will be used (blocks AB and BA). Once an eligible patient gives his informed consent, he will be given a specific, unique identifier. The statistician will monitor the enrollment process.

### Data management

During the study, the investigators will regularly fill in the case report forms (CRF). Database management and quality control for the study will be performed by an independent statistician. Structured data elements from the CRFs will be entered into the database and reviewed using double data entry for verification. Information entered into the database will be systematically checked and evident errors will be corrected. All of the omissions and/or questions concerning the data will be discussed with the investigator in order to preserve the completeness of the database.

### Statistical analysis

Baseline demographic characteristic and clinical variables will be summarized for each arm of the study. Continuous data will be presented as arithmetic means and SD for normally distributed variables or as medians and inter-quartile ranges (25 to 75th) for abnormally distributed variables. Normality will be tested by the Shapiro-Wilk test.

Comparison of two groups will be based on parametric Student’s two-sample *t*-test, or Cochran-Cox test or non-parametric Mann–Whitney test as appropriate. Student’s paired *t*- or Wilcoxon’s test will be used to compare continuous variable differences between baseline and the end of observation period.

Categorical data will be given as absolute and relative frequencies (percentages). The differences in proportions between them will be examined using the chi-square - test with Yates’s correction or Fisher’s exact test as appropriate.

A logistic regression model will be used to identify independent predictors of response to therapy. For the longitudinal analysis, curves of cumulative probability of events will be constructed according to the Kaplan-Meier method, and the cumulative events rates will be compared by the log-rank test. A Cox proportional hazards regression analysis will be performed to assess for any potential influence of covariates. The assumptions of the Cox proportional hazards model will be checked by visual inspection of the log-log survival function by time curve.

Due to the possibility of the treatment crossovers, all major treatment comparisons between the randomized group in this trial will be performed according to both the intention-to-treat and the per protocol principles.

All null hypothesis will be two-tailed with a 0.05 type I error rate.

Statistical analysis will be performed using the SAS 9.2 statistical package (SAS Institute Inc., Cary, NC, USA).

The investigation conforms with the principles outlined in the Declaration of Helsinki [21]. The local ethics committee (that is the Ethics Committee of the Institute of Cardiology, Warsaw, Poland) has approved the study protocol including the written informed consent form (approval number: IK-NP-0021-47/1378/13).

## Discussion

Based on the current knowledge, the authors assume that the *rhythm control* strategy would be at least as good as the *rate control* strategy in terms of BiVp%. The achievement of SR and satisfactory BiVp% in the *rhythm control* group may strengthen the underestimated position of cardioversion in the group of HF patients on CRT with concomitant permanent AF.

It may seem disputable whether the CRT patients with permanent AF will resume and maintain SR. As has been already mentioned, HF and AF are diseases that can negatively influence each other. In at least some of the patients, an occurrence of AF seems to be a marker of HF severity. We hypothesize that restoration of sinus rhythm, which would enhance the performance of CRT and enable better treatment of HF, may have beneficial effect on AF progression. Based on the evidence of the SR resumption phenomenon [13] and the study by Turco *et al*., this is highly probable. In the latter work it has been proven that - by means of a single cardioversion - SR is resumed in 82% of the patients and maintained for a 1 year in about 60% of the former AF-CRT patients [14]. We assume that the possibility of additional cardioversions and standard use of the amiodarone will elevate this percentage. Additionally, the results of the PVI isolation from the PABA-CHF trial in HF patients with AF are also appealing bearing in mind the initial differences between the populations of the PABA-CHF and our study (that is the lack of CRT in the PVI subgroup and paroxysmal character of the AF in half of the patients). After a 6-month observation period, 88% and 71% of patients who underwent PVI were free from AF depending on whether they received or did not receive antiarrhythmic agents [18].

The authors are fully aware that during the progression of HF, irreversible fibrous changes within the atria occur which promote AF and its maintenance [22], so it is unlikely that the proposed treatment strategy would be effective in all of the patients included. Hence, it seems crucial not only to establish the efficacy of proposed treatment strategy of rhythm control but also to define the subgroup of patients who would benefit the most from this strategy. An attempt to perform preliminary analysis of the non-responders as well as the ultra-responders would be made in the trial.

The standard use of amiodarone in both of the groups may seem disputable in the potential complications of this treatment and limited evidence of its efficacy. However, following the literature the use of amiodarone should facilitate sinus rhythm maintenance in the rhythm control group [1, 23, 24]. The use of the drug in the *rate control* group arises from its beneficial effect on AV node in this group of patients [1]. Moreover, it makes two of the treating arms more consistent. Following the latest ESC guidelines on management of AF [1], amiodarone is commonly used in patients with AF and depressed left ventricle ejection fraction and its potential adverse events, occurring in up to about 10% of patients according to the latest RCT comparing amiodarone with dronedarone [24], will be carefully monitored, including regular TSH measurements, QT evaluation and occurrence of arrhythmias at baseline visits and during the follow-up period.

One of the limitations of the trial is the number of the participants which surely has an impact on the statistical strength of the study. It would enable the researchers to assess the efficacy of the two treatment strategies on the primary endpoint only. The statistical difference within the clinical secondary endpoints of this study will be difficult to achieve. However, the lack of sufficient data about the CRT in the patients with concomitant AF necessitates the undertaking of an initial pilot study upon this issue.

The incoming results of the Pilot-CRAfT would enable the authors to prepare a parallel larger, well-designed multi-center study assessing the influence of both treatment strategies on ‘harder’ clinical endpoints, such as mortality and morbidity, as well as further analysis of the factors determining the response to the rhythm control strategy. The assessment of the efficacy of the rhythm control strategy in CRT and AF patients and the identification of the subgroup of patients who benefit from it the most would create an alternative to the currently recommended rate control strategy which has some considerable limitations.

Whatever the results of the trial, performing a prospective, randomized study on CRT patients with concomitant persistent forms of AF would finally provide strong scientific evidence data about the efficacy of CRT in this specific subgroup of patients. This urgently needed data has been long awaited.

## Trial status

Ongoing - currently recruiting.

## Abbreviations

6MWT: six-minute walk test
AF: atrial fibrillation
AVJA: atrioventricular junction ablation
BiVp%: effectively biventricular paced captured beats
BR: breathing reserve
CPX: cardiopulmonary exercise test
CRF: case report form
CRT: cardiac resynchronization therapy
EEC: external electrical cardioversion
EF: ejection fraction
ESC: European Society of Cardiology
SR: sinus rhythm
HF: heart failure
HRR: heart rate reserve
MLHFQ: Minnesota Living with Heart Failure Questionnaire
NYHA: New York Heart Association
PVI: pulmonary vein isolation
RCT: randomized controlled trial
SD: standard deviation
SR: sinus rhythm
TSH: thyroid-stimulating hormone
peak VCO_2_: peak carbon dioxide exhalation
peak VO_2_: peak oxygen uptake
VE: maximal minute ventilation.
